# Checkpoint Kinase 2 (CHEK2) Mutation in Renal Cell Carcinoma: A Single-Center Experience

**DOI:** 10.15586/jkcvhl.2018.101

**Published:** 2018-04-18

**Authors:** Joanna Huszno, Zofia Kołosza

**Affiliations:** 1 Outpatient Clinic, Maria Skłodowska-Curie Memorial Cancer Center and Institute of Oncology, Gliwice, Poland; 2 Biostatistics Unit, Maria Skłodowska-Curie Memorial Cancer Center and Institute of Oncology, Gliwice, Poland

**Keywords:** checkpoint kinase 2 mutation, CHEK2 mutation, renal cell carcinoma, retrospective study, second neoplasm

## Abstract

Renal cell carcinoma (RCC) occurs in sporadic and heritable forms. Genetic mutations have been identified as risk factors in 1–2% of RCC. The aim of this study was to evaluate I157T and CHEK2*1100delC mutations of checkpoint kinase 2 (CHEK2) gene in RCC. Medical records of 40 clear cell RCC patients who had genetic tests and consultation at the Genetic Outpatient Clinic, Maria Skłodowska-Curie Memorial Cancer Center and Institute of Oncology, Gliwice Branch, Poland, were reviewed retrospectively. Mutation profile was assessed by ASA-PCR and RFLP-PCR techniques. Only three female patients had CHEK2 mutation (I157T). No CHEK2*1100delC was observed in any of the patients. These tumors were N0, and two were Grade 3. One showed capsular infiltration. No blood vessel infiltration or metastases was observed. Overall, RCC from patients with CHEK2 mutation did not display any special characteristics when compared with those without the mutation. While no association between CHEK2 mutation and RCC could be established, all three patients with CHEK2 mutation developed second neoplasms many years after first diagnosis. Further studies, especially regarding CHEK2 mutation as a predictive factor for second neoplasm in RCC patients, are warranted.

## Introduction

Renal cell carcinoma (RCC) accounts for about 4% of all adult malignancies. RCC occurs in both sporadic and heritable forms (1). Genetic mutations have been identified as the cause of inherited cancer in 1–2% of RCC. Epidemiologic data suggest that a family history of RCC is a risk factor for the disease (2). The best-known hereditary cancer predisposition syndrome that leads to clear cell RCC is von Hippel–Lindau (VHL) disease (3, 4). Familial clear cell RCC is the most frequently diagnosed condition as a consequence of aberrations in VHL gene (2). Apart from *VHL*, mutations of *MET, FLCN, TSC1, TSC2, FH,* and *SDH* genes also are risk factors for the development of RCC (5, 6).

The human tumor suppressor gene checkpoint kinase 2 (CHEK2), located on chromosome 22q12.1, contains 14 exons (7). The gene encodes protein kinase Chk2 (cell-cycle-checkpoint of proteins kinase 2), which is crucial for the maintenance of genome integrity and the regulation of G2/M cell-cycle-checkpoint (8). The Chk2 enzyme plays a crucial role in ensuring accurate DNA repair in response to. CHEK2 also acts as a tumor suppressor by promoting genomic stability, enabling DNA repair, and inducing apoptosis (7). In response to DNA damage, *CHEK2* is activated via ATM-dependent pathway and phosphorylates several substrates such as p53, BRCA1, CDC25A, and CDC25C. CHEK2 mutation has been reported in breast cancer (8–15), colorectal cancer (16, 17), malignant melanoma (18), and bladder cancer (17, 19). Especially, the polymorphic variant of *CHEK2* gene (1100delC) is associated with increased risk of breast, prostate, colorectal, and thyroid cancers (9, 10, 20), whereas the missense variant I157T, in addition to the above cancers, is known to increase the risk of kidney cancer, ovarian adenocarcinoma, and borderline ovarian cancers (16, 21).

Although sparse, the available data suggest a role for CHEK2 in RCC. Trubicka et al. showed that the constitutive mutation I157T in *CHEK2* gene is responsible for the development of ccRCC (22). Ge et al. reported that a rare variant of CHEK2, rs17879961, was associated with decreased risk of renal cell cancer (23). A study by Ghatalia et al. (24), which compared intra-patient kinase gene expression between RCC and matched normal kidney samples, identified CHEK2 as one of the top 10 overexpressed kinases in metastatic RCC, suggesting a pathological role of activated CHEK2 in RCC. The aim of this retrospective study was to further evaluate the role of I157T and CHEK2*1100delC mutations of *CHEK2* gene in ccRCC.

## Material and Methods

Medical records of 40 ccRCC patients who were diagnosed and/or treated at the Outpatient Clinic, Maria Skłodowska-Curie Memorial Cancer Center and Institute of Oncology, Gliwice Branch, Poland, were reviewed. This was done according to the national guidelines. All patients signed written informed consent allowing the use of their biological material for clinical research. The patients had genetic tests and consultation at the clinic. The primary reason for these patients seeking genetic counseling was a history of cancer in the family. Mutation profile was performed as per the standardized procedure of the clinic. In brief, DNA was isolated from 10 mL peripheral blood sample. Status of CHEK2*1100delC and I157T mutations was assessed by ASA-PCR and RFLP-PCR techniques, respectively. In each amplification, both positive and negative controls were used. The 1100delC is a rare variant that leads to premature protein truncation. The relatively common missense variant I157T is the result of the substitution of isoleucine 157 by threonine.

The following inclusion criteria were applied: microscopic confirmation of ccRCC; performance status ZUBROD 0–1; age above 18; and renal (creatinine, urea, GFR), liver (transaminase and bilirubin levels), and bone marrow (hemoglobin concentration, white blood cell count, platelet count) functions within the normal range. Data on age at onset, co-morbid conditions, cigarette smoking, surgical procedure, TNM classification, histology, Fuhrman grade, history of cancer in family, and second neoplasm were gathered from hospital records and pathology reports.

Statistical analyses were carried out using STATISTICA 7 software. The qualitative features are presented as the percentage of occurrence and evaluated with Fisher test and Chi squared test with Yates correction. Differences were considered significant if the P-value was ≤ 0.05.

## Results

### Patient characteristics

The patient characteristics are shown in Table 1. All patients had nephrectomy due to kidney cancer, and all were confirmed to have ccRCC upon histopathologic examination. The median age of patients at diagnosis was 52.5 years (range from 25 to 78), and 60% of patients were ≥50 years old. The majority of the patients were women (75% women vs. 25% men, P = 0.159; Table 1). Co-morbid conditions were observed in 55% of patients. The predominant co-morbidities were cardiovascular diseases (35%) and diabetes (13%). None of the patients had a history of viral diseases. Cigarette smoking was reported in 32.5% of patients.

**Table 1:** Clinical characteristics of the kidney cancer patients.

Factors		n	%
Gender	Women Men	30 10	75 25
Age	≥50 <50	24 16	60 40
Co-morbid condition	Yes No	22 18	55 45
Diabetes	No Yes	35 5	87.5 12.5
Cardiovascular diseases	No Yes	26 14	65 35
Viral diseases	No Yes	40 0	100 0

### Mutation and tumor characteristics

The mutation status and tumor characteristics are summarized in Table 2. Only three patients had CHEK2 mutation (I157T), and all were females. CHEK2*1100delC was not observed in any of the patients. These tumors were N0 and less than 10 cm in size. Two were Grade 3, and one showed capsular infiltration. No blood vessel infiltration or metastases was observed. Overall, RCC from patients with CHEK2 mutation showed no special characteristics when compared with RCC from patients without CHEK2 mutation.

**Table 2:** Mutation and histological characteristics of the kidney cancer patients.

Factors		n	%	CHEK2 I157T (%)
Clinical staging nodes	N0 N1-N3	39 1	97.5 2.5	3 (100) 0
Tumor size	T<10 cm T>10 cm	39 1	97.5 2.5	3 (100) 0
Grade G	G1+2 G3	34 6	85 15	1 (33.3) 2 (66.7)
Blood vessels infiltration	No Yes Missing	36 3 1	90 7.5 2.5	3 (100) 0 0
Adrenal gland metastases	No Yes Missing	37 2 1	92.5 5 2.5	3 (100) 0 0
Capsule infiltration	No Yes Missing	33 6 1	82.5 15 2.5	2 (66.7) 1 (33.3) 0

### Family history and secondary neoplasms

Cancer in family history was reported in 70% of patients (Table 3). The predominant was colorectal cancers (35%), followed by breast cancer (25%), gynecological cancers (15%), and renal cell cancer (12.5%). Our results showed no association between CHEK2 mutation and cancer in the family. In all patients with *CHEK2* mutation, second neoplasms developed many years after the first diagnosis: breast cancer 15 years post-nephrectomy; renal cancer 2 years post-nephrectomy; and meningioma 9 years post-nephrectomy.

**Table 3:** The presence of cancer in family history.

Type of cancer		n	%
History of cancer in family:	Yes	28	70
Colorectal cancer	Yes	14	35
Breast cancer	Yes	10	25
Lung cancer	Yes	6	15
Gynecological cancer	Yes	6	15
Renal cancer	Yes	5	12.5
Larynx cancer	Yes	2	5
Stomach cancer	Yes	0	0
central nervous system (CNS)	Yes	0	0

## Discussion

In this retrospective study, we analyzed the presence of *CHEK2* mutation and its relationship with cancers in family history of patients with ccRCC. Interestingly, there were more women than men in the study group, a factor that is contradictory to the established norm that RCC is more prevalent in men than in women. The reason for this discrepancy is not clear. However, given that the study population is patients seeking genetic counseling because of a history of cancer in the family, it appears to confirm the notion that men are less willing than women to participate in regular screening processes. Overall, *CHEK2* mutation (variant I157T) was detected in only three patients. CHEK2 mutation neither appeared to have conformed any special characteristics to these tumors nor had a role in family history.

The putative role of CHEK2 mutation has been reported in other cancers. For example, Cybulski et al. reported that while the I157T *CHEK2* mutation increases the risk of colorectal cancer in Polish population, truncating mutations may be associated with a low or no risk (16). Similar results (an increased risk of sporadic and familiar colorectal cancer in patients with *CHEK2* I157T mutation) were reported by Kilpivaara et al. (17). The increased risk of breast cancer in patients with *CHEK2* mutation is supported by many studies (9, 11, 12). *CHEK2* missense variant I157T is found to be associated with a 1.5-fold risk of breast cancer (13), and CHEK2*1100delC heterozygosity is associated with a three-fold risk of breast cancer in women in the general population (14). A meta-analysis conducted in 26,000 patients and 27,000 controls showed that CHEK2*1100delC increases the risk of breast cancer three-to five-fold. The risk of breast cancer at age 70 years in CHEK2*1100delC heterozygotes is almost as high as that for *BRCA1* and *BRCA2* mutation heterozygotes (15). In some meta-analyses, CHEK2*1100delC heterozygotes have a two-fold risk of malignant melanoma (18).In addition, Złowocka et al. have found that CHEK2 mutations increase the risk of bladder cancer (19). In our previous study, breast cancer patients with CHEK2 mutation were characterized by older age, history of gastric cancer in family, and lower stage of disease (10). Pertinent to RCC, a relationship with CHEK2 mutations has been reported previously (9, 25). Specifically, the missense variant I157T was associated with an increased risk of kidney cancer (9). In the current study, no such relationship could be established.

The caveat of this study is that this is a single-center retrospective study with only 40 patients. However, the most important finding is that all three patients with CHEK2 mutation developed second neoplasms. Thus, the study provides a rationale for further exploration of the role of CHEK2 mutation in RCC, especially its utility as a predictive factor for secondary neoplasms.

## Conflict of Interest

The authors declare no potential conflicts of interest with respect to research, authorship, and/or publication of this article.
